# Strong and ductile AZ31 Mg alloy with a layered bimodal structure

**DOI:** 10.1038/s41598-019-41987-4

**Published:** 2019-04-01

**Authors:** Xuan Luo, Tianlin Huang, Yuhui Wang, Yunchang Xin, Guilin Wu

**Affiliations:** 1 International Joint Laboratory for Light Alloys (MOE), College of Materials Science and Engineering, Chongqing University, Chongqing, 400044 China; 2 National Engineering Research Center for Equipment and Technology of Cold Strip Rolling, Yanshan University, Qinhuangdao, 066004 China

## Abstract

AZ31 Mg alloy was processed by accumulative roll-bonding (ARB) and hot rolling (HR), respectively, followed by annealing. Layered bimodal structures characterized by an alternative distribution of fine-grained layers and coarse-grained layers were obtained in the ARB samples, while mixed bimodal structures were achieved in the HR samples. The ARB samples have superior combinations of high strength and good elongation compared to the HR samples, indicating a clear effect of layered bimodal structures on mechanical properties of the alloy. The strength of the ARB samples is related to the grain size; while the ductility is attributed to the activity of non-basal slip and the strong backstress.

## Introduction

Ultrafine-grained (UFG) materials have recently drawn great interest due to their improved strength compared with their coarse-grained (CG) counterparts^1,2^. However, the ductility of the UFG materials is normally limited. Previous studies have reported numbers of strategies to improve the ductility, i.e., severe plastic deformation (SPD)^3,4^, nanotwins^5^, bimodal microstructures^6,7^, and gradient structures^8,9^. However, most of these observations of the bimodal structures were found in cubic crystal structured metals.

Mg alloys, with a hexagonal close-packed crystal structure, have various advantages, i.e., high specific strength and lightweight. However, the poor ductility of Mg alloys limits their applications. The deformation behaviour and mechanical properties of Mg alloys are highly dependent on the grain size^10–12^, and a reduced twinning activity was always observed in small-sized Mg^13^. A bimodal grain structure composing fine-grains and coarse-grains in micrometer scale was often observed in SPD processed Mg alloys^14–17^, which was reported in some cases undesirable for mechanical properties^18^. Pioneering studies have shown that pure Ti with a heterogeneous lamella structure can unite UFG strength and coarse-grain ductility^19^. Such combinations of high strength and good ductility were also demonstrated recently in an interstitial free (IF) steel^20^ and a Mn steel^21^. Therefore, the present study is aimed to produce a layered bimodal grain structure in Mg alloys and address the effect of bimodal structures on mechanical properties.

## Results

### Microstructures

**Figure 1:**
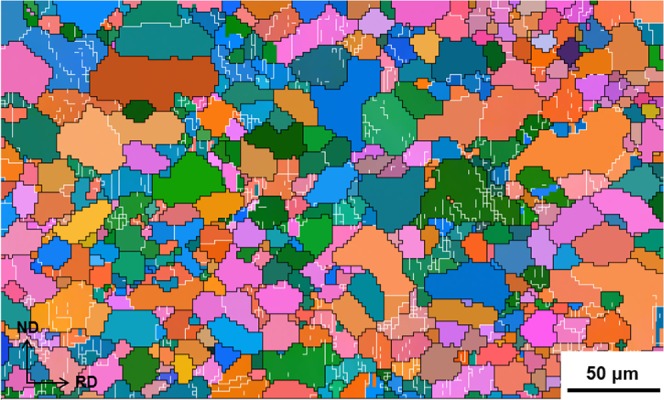
EBSD map of the starting material. In the EBSD map, white lines represent misorientation angles 2°–15°, and black lines ≥15°.

**Figure 2:**
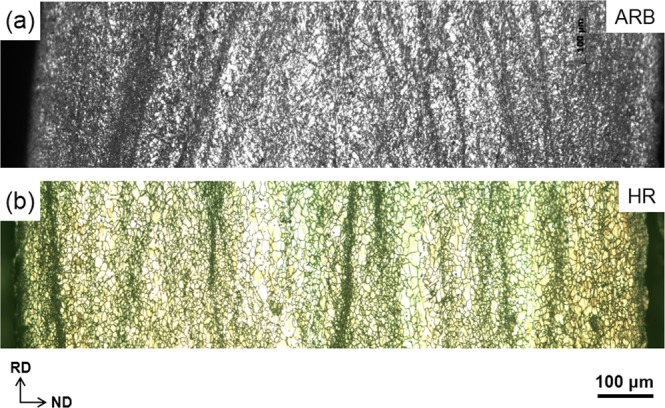
Optical micrographs of the ARB (**a**) and HR (**b**) sheets.

The CG sheets have a coarse structure with an average grain size (*d*_*av*_) of 10.6 μm (Fig. 1). ARB and HR greatly reduced the grain size although the microstructures are quite heterogeneous (Fig. 2(a,b)). The presence of fine grains and coarse grains suggests that dynamic recrystallization (DRX) occurred during rolling, which was frequently reported in Mg alloys^15,22^. The coarse grains are dynamically deformed and fine-grains are produced by DRX.

**Figure 3:**
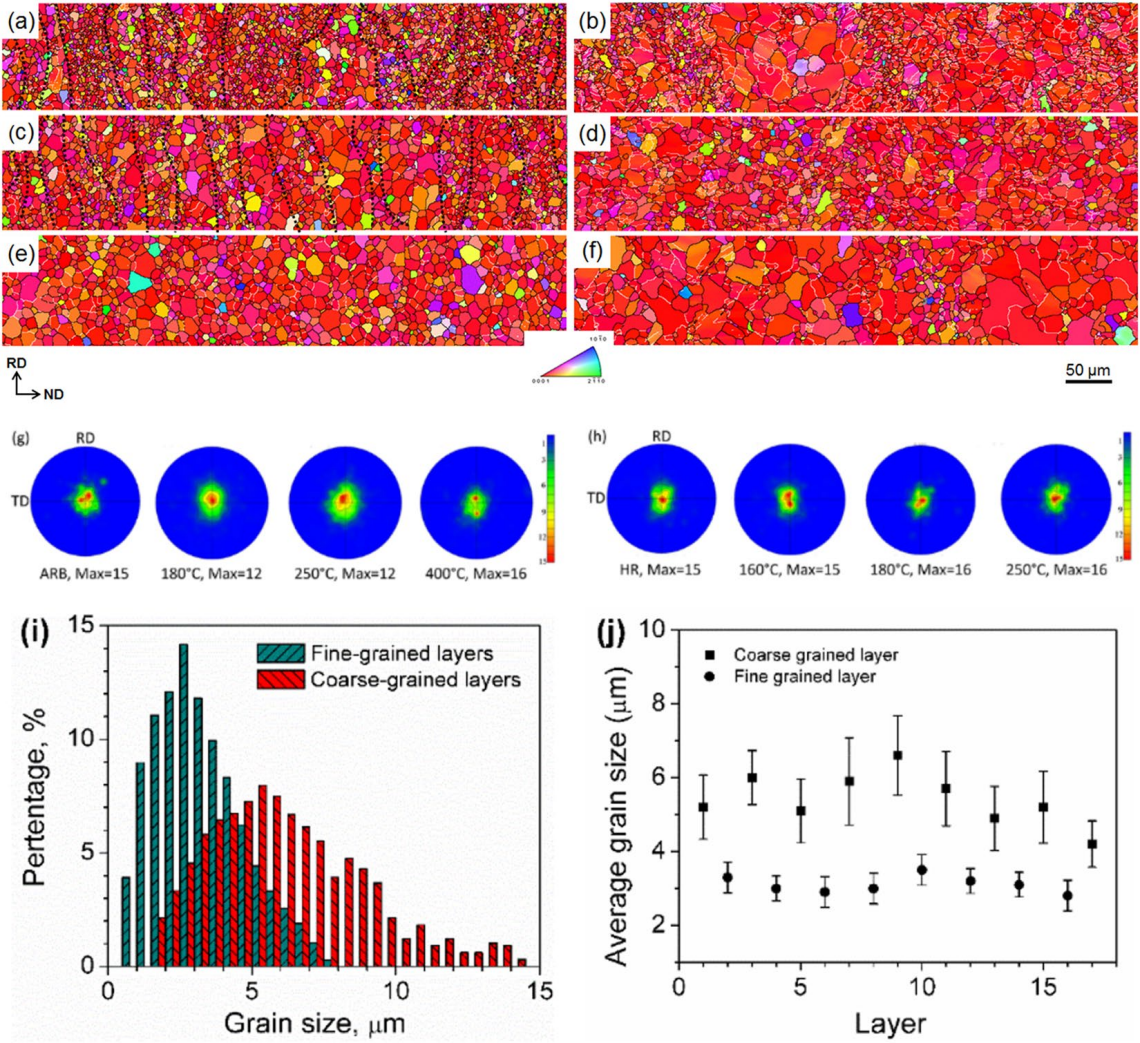
EBSD inverse pole figure (IPF-ND) maps of ARB samples annealed at (**a**) 180 °C, (**c**) 250 °C, (**e**) 400 °C and HR samples annealed at (**b**) 160 °C, (**d**) 180 °C, **(f**) 250 °C. (**g**) and (**h**) are {0002} pole figures of the annealed ARB and HR samples, respectively. (**i**) The grain size distributions of fine-grained and coarse-grained layers and (**j**) *d*_*av*_ of individual layers through the thickness of the 250 °C annealed ARB sample. In (**a**) and (**c**), black dot lines indicate the interfaces between fine-grained and coarse-grained layers.

Figure 3(a,c and e) are EBSD maps of the annealed ARB samples. The annealed samples are recrystallized and the grain size increases with increasing temperature. The microstructures of the samples annealed at 180 °C and 250 °C are heterogeneous, consisting of alternatively distributed fine-grained and coarse-grained layers. The fine-grained layers are caused by DRX in deformation bands during hot rolling^23^, while coarse grains are thought to be caused by static recrystallization during annealing, which will be studied in our future work. The layers are approximately 20–50 μm thick. For the 180 °C annealed sample, the *d*_*av*_ is 2.5 μm and the *d*_*av*_ for the fine-grained and coarse-grained layers are 1.9 μm and 3.8 μm, respectively. For the 250 °C annealed sample, the fine-grained and coarse-grained layers are more apparent. Figure 3(i) shows the grain size distribution of fine-grained and coarse-grained layers. A bimodal distribution of grain sizes is seen, indicating a bimodal structure in the sample. Figure 3(j) shows the distribution of *d*_*av*_ of the layers through about half thickness of the sample. The *d*_*av*_ for the fine-grained and coarse-grained layers is 2.8 μm and 5.2 μm, respectively, corresponding well to the bimodal grain size distribution, which gives rise to a value of *d*_*av*_ = 3.4 μm. After annealing at 400 °C, the sample develops a relatively homogeneous microstructure with a *d*_*av*_ of 5.2 μm. All grains grow during annealing so that the difference in sizes disappeared after annealing at 400 °C. Although the 180 °C and 250 °C annealed ARB samples have bimodal microstructures, these structures are different from other bimodal microstructures with heterogeneous grain structures in Mg alloys^14–17^. The fine grains and coarse grains form layers, similar to the laminated composite. Therefore, these microstructures are described as layered bimodal structures.

As seen in Fig. 3(b,d and f), the microstructures of the HR samples after annealing are generally quite heterogeneous containing fine grains and coarse grains; and some grains are even larger than 30 μm. The microstructure is very similar to an AZ31 processed by repeated rolling and annealing using a small pass reduction rate^24^. The average grain sizes increase with increasing temperature, being 2.8, 3.4 and 5.8 μm, respectively. In general, the structures of the annealed HR samples are composed of randomly mixed fine and coarse grains, which hardly form regular layers. So, these structures are described as mixed bimodal structures.

### Mechanical properties of characteristic structures

**Figure 4:**
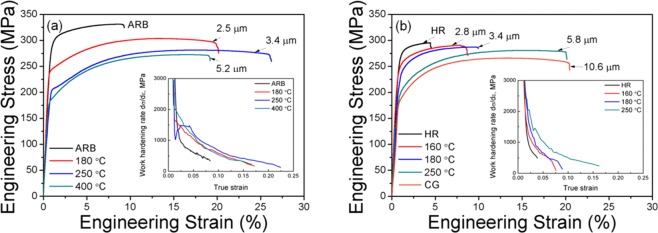
Tensile stress-strain curves of the (**a**) ARB and (**b**) HR samples. Insets in the figures show the work-hardening rate curves.

Figure 4 shows the engineering stress-strain curves of the ARB and HR samples. The curve of the CG sample is also included in Fig. 4(b). The low yield strength (170 MPa) and a large elongation (20%) of the starting sample are typical for CG AZ31 alloys. After ARB and HR processing, the yield strengths are significantly improved to 288 MPa and 267 MPa, and the elongations decreased to 8% and 4%, respectively, which is similar to previously observed in deformed Mg alloys^25,26^.

For the ARB sample, the yield strength decreases after annealing at 180 °C, yet keeping a high level of 240 MPa. However, the elongation of the sample increases significantly to 21%, close to that of the CG sheet. After annealing at 250 °C, the yield strength decreases to 203 MPa and the elongation increases to a maximum value of 26%. After annealing at 400 °C, the yield strength continuously decreases; meanwhile the elongation also decreases, similar to those of the CG sheet. The work-hardening rate curves (inset of Fig. 4(a)) show that the annealed samples show up-turns or plateaus in these curves, which demonstrates that the alloy has an extra work hardening after yielding.

For the HR samples, the strengths decrease and the elongations increase gradually with annealing temperatures and the work-hardening rates continuously decrease after yielding (inset of Fig. 4(b)), which are similar to previous observations^27^. After annealing at 250 °C, the yield strength and elongation of the sample are close to those of the CG material.

**Figure 5:**
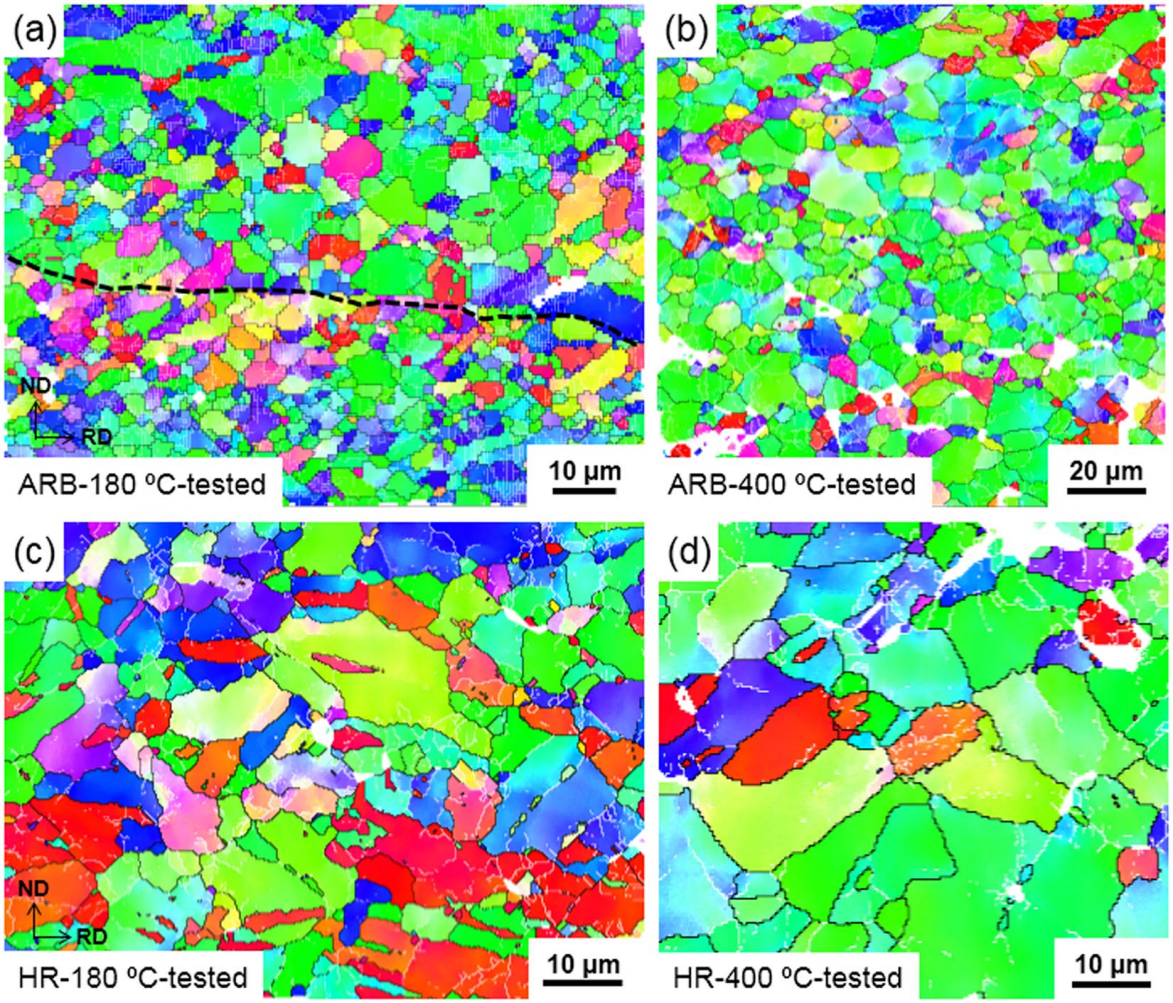
EBSD maps of (**a**) 180 °C and (**b**) 400 °C -ARB samples after tensile test and (**c**) 180 °C and (**d**) 400 °C -HR samples after tensile test.

**Figure 6:**
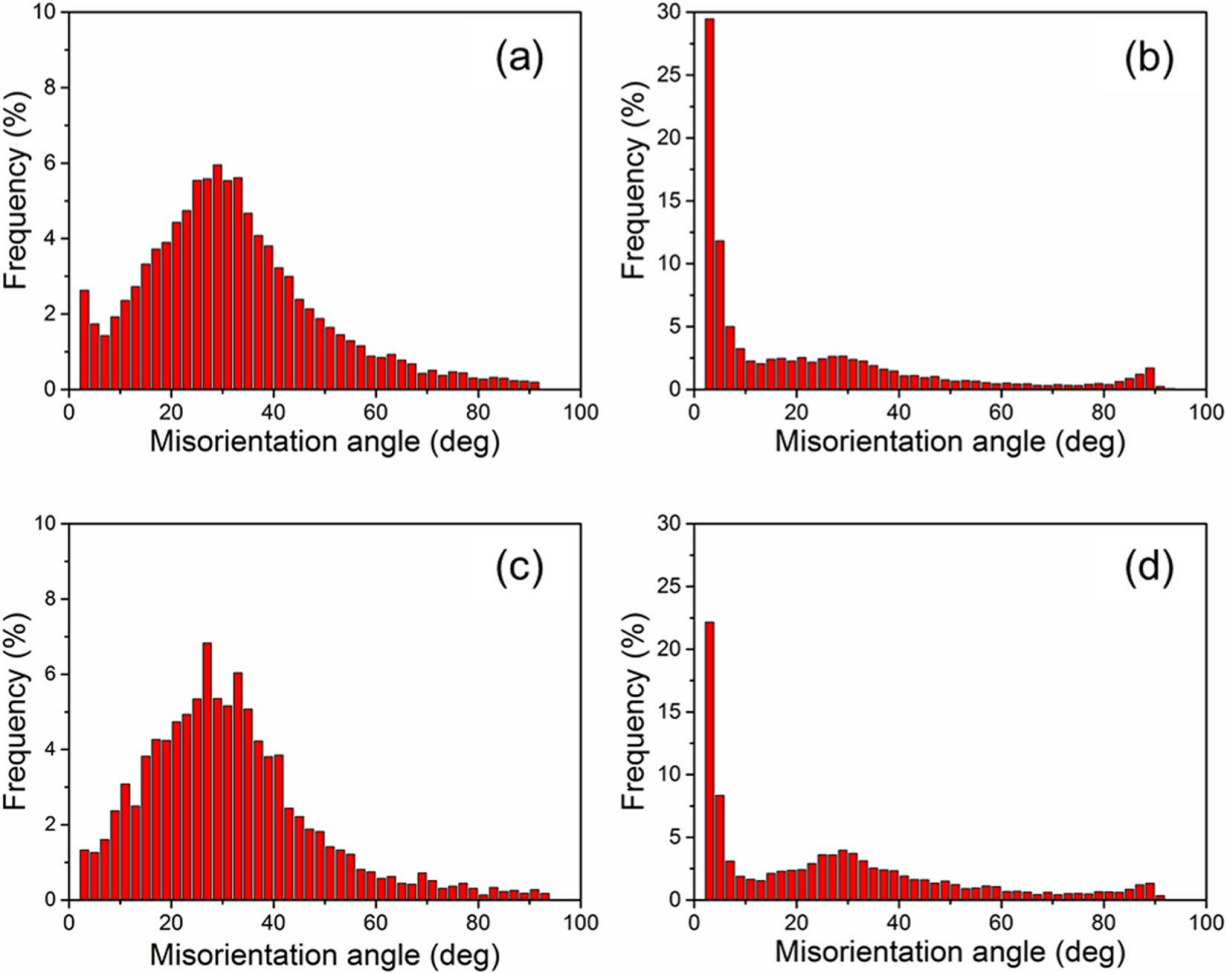
Grain boundary misorientation distributions of ARB samples (**a**) 180 °C before tensile test, (**b**) 180 °C after tensile test, (**c**) 400 °C before tensile test, (**d**) 400 °C after tensile test.

**Figure 7:**
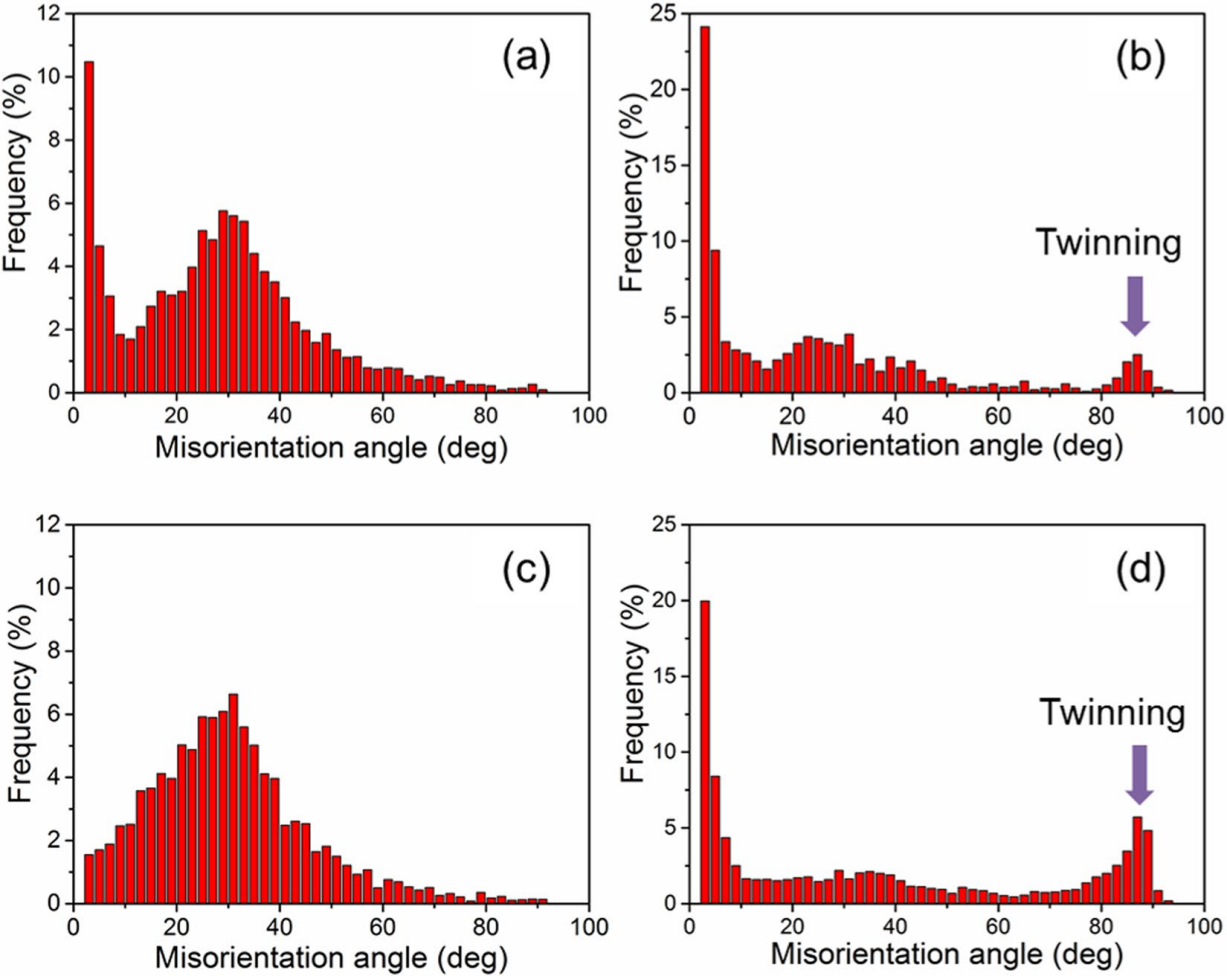
Grain boundary misorientation distributions of HR samples (**a**) 180 °C before tensile test, (**b**) 180 °C after tensile test, (**c**) 400 °C before tensile, (**d**) 400 °C after tensile test.

Figure 5 shows the EBSD maps of ARB and HR samples after the tensile test. In Fig. 5(a), the interface between a coarse-grained layer and a fine-grained layer is indicated by a black dashed line. Many low angle boundaries were found in both bimodal structured samples. Note the activation of twins in the HR samples with coarse grains. Figures 6 and 7 show the distributions of grain boundary misorientation for the ARB and the HR samples before and after tensile tests, respectively. It is seen that the extension twin activity was very limited in the ARB samples, while large number of extension twins were activated in the HR samples during the tensile tests. The reason for this difference is that most of the grains in the ARB samples are relatively small, whereas very coarse grains are present in the HR samples. It has been discussed that fine grains prohibit the extension twinning^13,14^, while extension twinning prevails in coarse grains during tensile deformation of AZ31 alloy with a strong basal texture.

**Figure 8:**
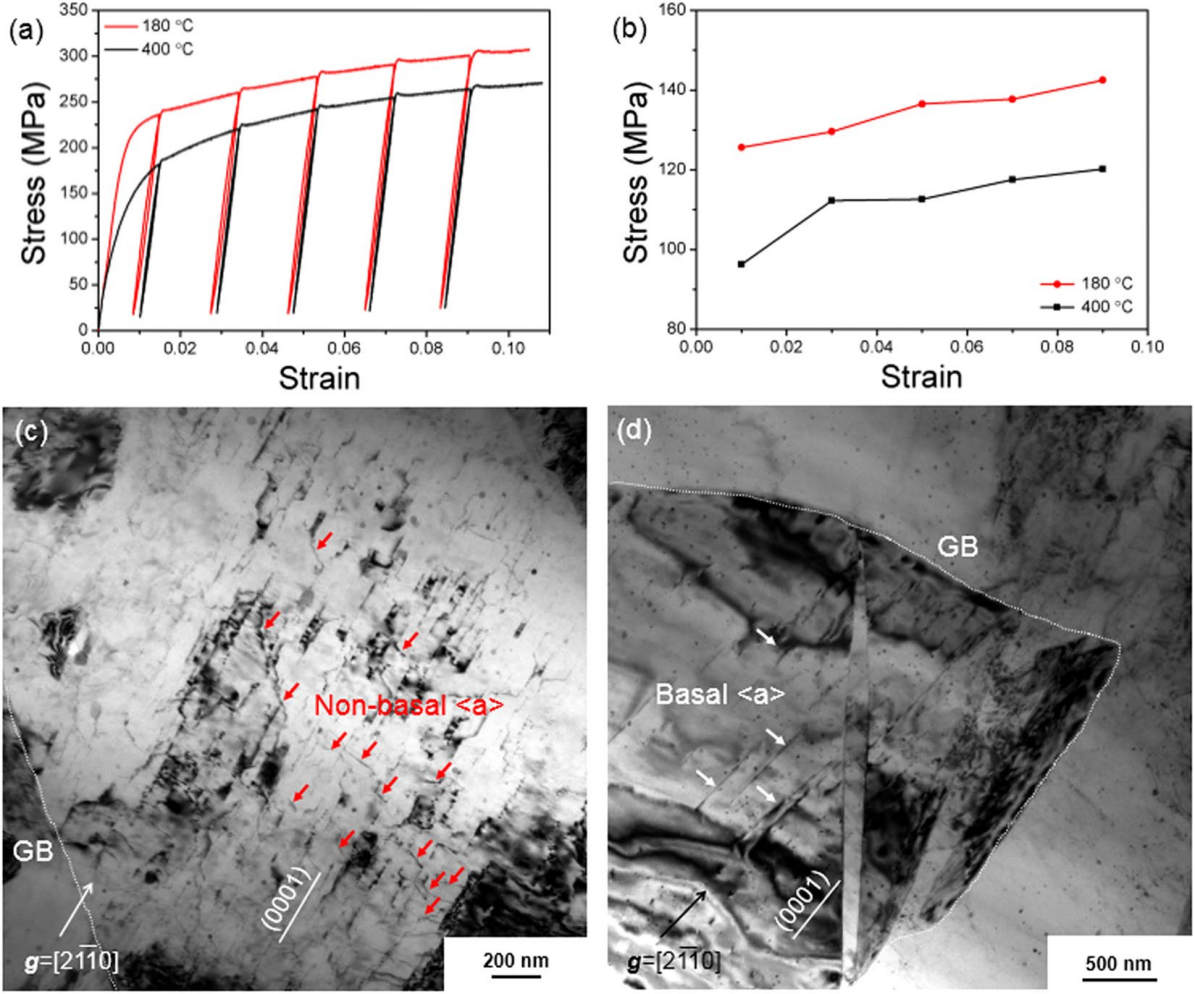
(**a**) Loading-unloading-reloading tensile tests of the 180 °C and 400 °C annealed ARB samples; (**b**) back-stresses determined from the curves in (**a**). (**c**) and (**d**) TEM images showing the microstructures for the 180 °C (**c**) and 400 °C (**d**) annealed and tensile-tested ARB samples.

Figure 8(a) shows the results of LUR tests of the ARB samples annealed at 180 °C and 400 °C, and stress-strain hysteresis loops are observed in these samples. It is seen that the 180 °C annealed sample shows wider LUR loops than the 400 °C annealed sample. From the LUR loops, backstresses were determined and are shown in Fig. 8(b). It is seen that the 180 °C annealed ARB sample has larger backstresses than the 400 °C annealed sample. Figure 8(c,d) show TEM images of the samples after tensile testing. Dislocations are observed in the microstructure of the tested samples. High density of non-basal dislocations were observed in the sample annealed at 180 °C. In contrast, mainly basal dislocations are seen in the sample annealed at 400 °C.

## Discussions

Comparing the stress-strain curves of the ARB and HR samples, it is seen that the yield strength of an ARB sample is close to that of an HR sample when they have a similar average grain size. The yield strength, therefore, is mainly related to the grain size, which follows the Hall-Petch equation. As shown in Fig. 4, the elongations of the ARB samples are much larger than those of the HR samples. For example, the 180 °C annealed ARB sample and the 160 °C annealed HR sample have similar average grain sizes and thus similar yield strengths, while the elongation of the ARB sample is almost twice larger than that of the HR sample. The ARB sample annealed at 250 °C shows a maximum elongation of 26%. Therefore, the ARB samples annealed at 180 °C and 250 °C have superior combinations of high strength and large elongation. It is also noticed that a yield point phenomenon appears in the stress-strain curve of the ARB sample with a grain size of 3.4 µm. Following a small Lüders deformation, the up-turn work hardening behaviour (inset of Fig. 4a) implies rapid activation and excessive multiplication of dislocations from supplementary slip systems.

The mechanical properties of Mg alloys are mainly influenced by the microstructure and texture. The present results showed that after ARB or HR and annealing, strong basal textures without basal poles splitting to the RD^9,23,24^ are developed in all samples, especially in the ARB samples, as shown in Fig. 3(g,h). Similar development of strong basal textures often has been observed in the rolled and annealed AZ31 alloys. Therefore, it is considered that the difference in elongation between the ARB and the HR samples is not due to the texture but caused by their different bimodal structures. The layered bimodal structure can be taken as a unique case of heterogeneous structures^28,29^, but is much more effective in producing strain hardening than the reported conventional heterogeneous structures, for example, bimodal structure^14–17^, gradient structure^30,31^ and harmonic structure^32^. The enhanced strain hardening in the layered bimodal structure is attributed to the high density of interlamellar interfaces, where dislocation can pile up and accumulate to enhance backstress hardening and dislocation hardening^19,28^.

The ARB samples annealed at 180 °C and 250 °C are characterized by layered bimodal structures. The coarse-grained layers should be softer and the fine-grained layers harder due to the Hall-Petch effect. During tensile deformation, the strain has to be continuous at the interlamellar interfaces, which leads to strain gradient near these interfaces. In the soft layers, geometrically necessary dislocations (GNDs) will be generated to accommodate the inhomogeneous deformation caused by the strain gradient^19,28^, while the hard layers remain elastic at a transition stage before macroscopic yielding, which generates a long-range backstress^28^ near the interfaces that will influence the further deformation. The soft layers cannot deform freely due to the strain constraint at layer interfaces. Note that the constraint effect between soft and hard layers together with the high yield stress induced by the small grain size and extra hardening associated with the yield point phenomenon may facilitate the activation of supplementary slip systems that have high CRSS. The activation of non-basal slip systems in the ARB samples is expected to enhance the accumulation and interaction of dislocations, which in turn enhances the work hardening and tensile elongation (Fig. 4a). Note that after macroscopic yielding the bask-stresses still exist due to strain partitioning caused by strain gradient between layers^28^. The backstresses also increase the work hardening of the samples, contributing to the enhancement of tensile elongation.

Such an effect of the backstress on the work-hardening was also demonstrated in the pure Ti^19^ and the IF steel^20^ with heterogeneous lamella structures. High strength and high ductility were also reported in an AZ91 alloy with a multimodal grain structure^33^, in which coarse grains were very elongated forming a mimetic layered bimodal structure locally. In addition, high strain-rate rolled (250 °C) AZ31 alloy showing high strength and ductility was reported with a heterogeneous structure of fine recrystallized grains and deformed matrix (yet recovery). These results also indicate the importance of heterogeneous structure for high ductility of Mg alloys^34^. The activation of non-basal dislocations in the 180 °C annealed ARB sample (Fig. 8(c)) is in agreement with the requirement of generation of GNDs in the coarse grains, and non-basal dislocations can better accommodate the c-axis strain^11^. Therefore, a good ductility can be achieved in the 180 °C annealed ARB sample. Consequently, a superior combination of high strength and large elongation is obtained in the 180 °C annealed ARB sample. In comparison, the layered bimodal structure feature of the ARB materials becomes un-distinguished after 400 °C annealing. Therefore, the extra work hardening due to the layered bimodal structure should be weak. The tensile elongation is reduced to a level similar to that of CG Mg alloys. HR samples have smaller elongations than the ARB samples, especially for the HR samples annealed at low temperatures. The lower elongations of the annealed HR samples are attributed to their mixed bimodal structures.

The cyclic loading-unloading tensile and compression tests for Mg alloys with fine or coarse grains have been discussed^35^. For Mg alloys with coarse grains, hysteresis loops were normally observed because of twinning during loading and thus de-twinning during unloading. As shown in Fig. 3, we prepared ARB samples with fine grains and HR samples with coarse grains. In addition, misorientation distributions of HR samples in Fig. 7 show that excessive extension twins were activated during the tensile tests. That is to say, if loading-unloading-reloading tests were performed on the HR samples, the hysteresis loops effect by twinning and de-twinning would cause large biases on the determined of backstresses by the method adopted in this study. However, for the ARB samples, the twinning activity during loading and the de-twinning activity during un-loading is very limited. The bias of twinning and de-twinning on the hysteresis loops can be neglected. Therefore, the determined backstresses mainly reflect the dislocation behaviours in the ARB samples. The loading-unloading-reloading tests cannot be applied to and compared directly with the ARB and HR samples due to different mechanisms. Herein, we only conducted the backstress analysis of the ARB sample to show the effect of layer bimodal structures.

**Figure 9:**
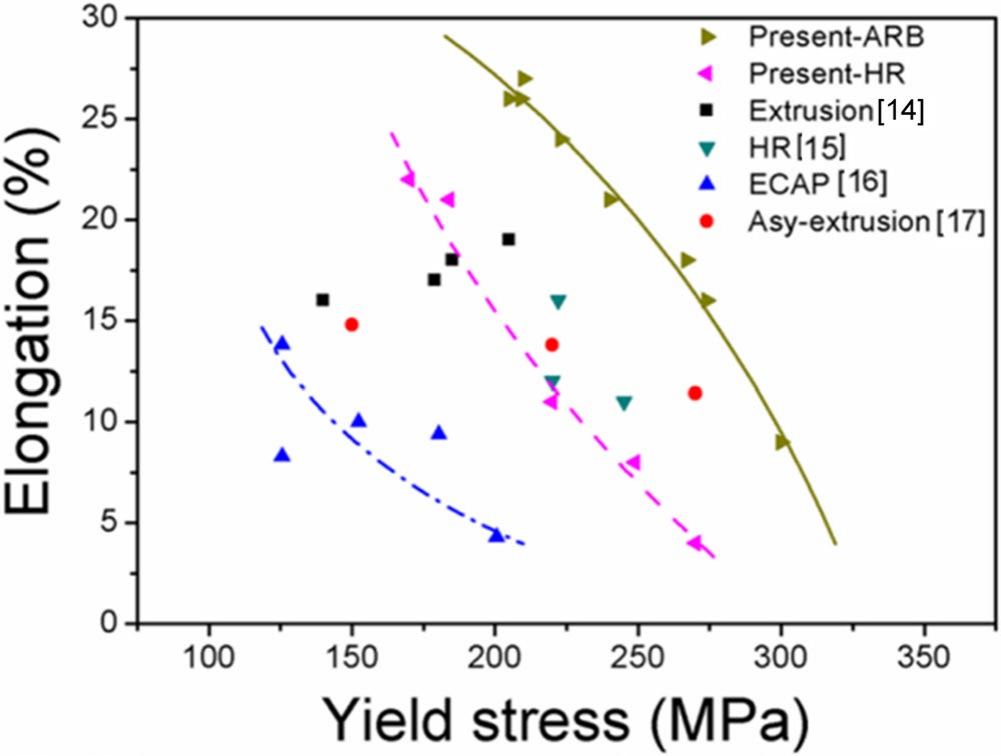
Plot of elongation versus strength of Mg AZ31 alloys.

Figure 9 shows the curves of the elongation versus yield strength obtained in the present study and from the literature. It is seen that the strength and elongation of the ARB samples in the present study are superior compared to other reported data with different microstructures, i.e., bimodal structures^14^, homogeneous fine-grained structure after HR^15^ and equal channel angular pressing (ECAP)^16^ and inhomogeneous structure by asymmetric hot extrusion^17^. The curve for the ARB samples shows a convex shape, which is opposite to the concave shape (also called the banana curve) for the HR samples. The convex shape is similar to that of the IF steel with heterogeneous layer and lamellar structures^20^, which also indicated that an extra work hardening originates from the structure of the alloy.

In summary, grain structure architecture was achieved in AZ31 Mg alloy by thermomechanical processing. Layered bimodal structures characterized by alternating fine-grained layers and coarse-grained layers were obtained by two-cycle ARB processing and subsequent annealing at 180 °C and 250 °C for 1 h. The AZ31 Mg alloy with layered bimodal structures has superior combinations of high strength and large tensile elongation (i.e., 240 MPa and 21%, 203 MPa and 26%). The strength is mainly related to the grain size and the elongation is caused by extra work-hardening produced by backstresses in the layered bimodal structures and non-basal slip in grains. The present work demonstrates a new method to optimize the mechanical properties of Mg alloys by producing layered fine and coarse grain structures even with a strong basal texture.

## Methods

### Material preparation

The material used in this study was commercial AZ31 Mg alloy. The material was hot rolled and annealed at 400 °C for 3 h to achieve fully recrystallized CG sheets. The sheets were processed by two comparative routes: accumulative roll-bonding (ARB)^36^ and hot rolling (HR). For the ARB processing, two 1-mm thick sheets were stacked and rolled to 50% reductions in one pass, cut into two halves, stacked and rolled 50% again. Before stacking, the sheet surfaces were degreased and wire brushed. For the HR processing, 4 mm thick sheets were rolled by 13 passes with 10% rolling reductions per pass. The rolling reductions of both routes were therefore 75% (a true strain of 1.39). For both processing routes, the sheets were pre-heated at 400 °C for 8 min before each pass, and the rollers were not lubricated or pre-heated. After rolling, samples were annealed at different temperatures for 1 h.

### Tensile test

Tensile specimens with gauge dimensions of 10 × 4 mm^2^ were cut from the processed sheets and tested with a strain rate of 4 × 10^−4^ s^−1^ at room temperature. During testing, the loading direction is parallel to the RD (RD, TD and ND represent the rolling direction, transverse direction and normal direction, respectively). Three tensile specimens were tested for each condition. In addition, loading-unloading-reloading (LUR) tensile tests^19^ were carried out.

### Microstructure characterization

Metallurgy specimens were prepared following the method in^13^ for optical observation. Samples were also characterized by using an Oxford AZtec electron backscatter diffraction (EBSD) detector attached to a Zeiss Auriga scanning electron microscope and a JEOL 2100 transmission electron microscope (TEM). EBSD specimens were electrochemically polished in the AC2 solution. TEM samples were cut from the RD-ND section of the uniformly elongated sections of tensile specimens, and then prepared by ion-milling after grinding to 100 μm thick. Based on the **g**·**b** criterion^37^, Burger vectors of dislocations were determined.

## Data Availability

The data required to reproduce these findings are available after request to the corresponding author.
